# Simple statistical models can be sufficient for testing hypotheses with population time‐series data

**DOI:** 10.1002/ece3.9339

**Published:** 2022-09-27

**Authors:** Seth J. Wenger, Edward S. Stowe, Keith B. Gido, Mary C. Freeman, Yoichiro Kanno, Nathan R. Franssen, Julian D. Olden, N. LeRoy Poff, Annika W. Walters, Phillip M. Bumpers, Meryl C. Mims, Mevin B. Hooten, Xinyi Lu

**Affiliations:** ^1^ Odum School of Ecology University of Georgia Athens Georgia USA; ^2^ Division of Biology Kansas State University Manhattan Kansas USA; ^3^ U.S. Geological Survey Eastern Ecological Science Center Athens Georgia USA; ^4^ Department of Fish, Wildlife, and Conservation Biology Colorado State University Fort Collins Colorado USA; ^5^ U.S. Fish and Wildlife Service Albuquerque New Mexico USA; ^6^ School of Aquatic and Fishery Sciences University of Washington Seattle Washington USA; ^7^ Department of Biology Colorado State University Fort Collins Colorado USA; ^8^ U.S. Geological Survey Wyoming Cooperative Fish and Wildlife Research Unit, Department of Zoology and Physiology and Program in Ecology University of Wyoming Laramie Wyoming USA; ^9^ Department of Biological Sciences Virginia Tech Blacksburg Virginia USA; ^10^ Department of Statistics and Data Sciences The University of Texas at Austin Austin Texas USA

**Keywords:** autoregressive, Etowah River, Konza Prairie, population ecology, regression, species abundance

## Abstract

Time‐series data offer wide‐ranging opportunities to test hypotheses about the physical and biological factors that influence species abundances. Although sophisticated models have been developed and applied to analyze abundance time series, they require information about species detectability that is often unavailable. We propose that in many cases, simpler models are adequate for testing hypotheses. We consider three relatively simple regression models for time series, using simulated and empirical (fish and mammal) datasets. Model A is a conventional generalized linear model of abundance, model B adds a temporal autoregressive term, and model C uses an estimate of population growth rate as a response variable, with the option of including a term for density dependence. All models can be fit using Bayesian and non‐Bayesian methods. Simulation results demonstrated that model C tended to have greater support for long‐lived, lower‐fecundity organisms (K life‐history strategists), while model A, the simplest, tended to be supported for shorter‐lived, high‐fecundity organisms (r life‐history strategists). Analysis of real‐world fish and mammal datasets found that models A, B, and C each enjoyed support for at least some species, but sometimes yielded different insights. In particular, model C indicated effects of predictor variables that were not evident in analyses with models A and B. Bayesian and frequentist models yielded similar parameter estimates and performance. We conclude that relatively simple models are useful for testing hypotheses about the factors that influence abundance in time‐series data, and can be appropriate choices for datasets that lack the information needed to fit more complicated models. When feasible, we advise fitting datasets with multiple models because they can provide complementary information.

## Graphical abstract

We demonstrate the utility of three simple statistical models for analyzing time‐series data of organismal abundances, using simulation and real‐world examples.

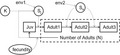

## 1 INTRODUCTION

Understanding factors that govern population size through time is a central theme in ecology, with a rich history of inquiry that spans theoretical and mathematical (Hassell, 1975; May, 1975; Turchin, 1990) to empirical and applied approaches (Beissinger & McCullough, 2002; Morris & Doak, 2002). Over the past decade, advances in state–space models have woven together a number of these different research lineages. For example, efforts have successfully incorporated density‐dependent population growth mechanisms into data‐driven statistical models of population time series while accounting for imperfect detection (Dail & Madsen, 2011; Hostetler & Chandler, 2015; Kanno et al., 2015; Zipkin et al., 2014). Model extensions account for excess zeroes due to immigration/emigration (Hostetler & Chandler, 2015) and simultaneous analysis of multiple populations, which facilitates viability analysis for less intensively sampled populations (Leasure et al., 2019; Wenger et al., 2017). Mark–recapture models have likewise been extended to hierarchical models in which demographic processes are the focus (Link & Barker, 2005), joint models of interacting species (Yackulic et al., 2018), and integrated population viability models (Saunders et al., 2018), among others.

Two fundamental challenges characterize these recent modeling advances: (1) they are data intensive, generally requiring additional sampling effort to estimate observation error, and (2) they are structurally complex, which puts them beyond the reach of many practitioners. The first point is the most important because many existing time series datasets lack the information needed to fit an observation model, rendering such approaches infeasible. However, the complexity of the modeling can be a barrier even when all requisite data are available. Most such models must be fit using custom‐coded Bayesian methods, often requiring weeks to months of development and troubleshooting. With large datasets, they may require considerable computational time to fit a single model, although recent advances have reduced this time (e.g., Yackulic et al., 2020). Much of this complexity is a necessary result of incorporating observation and sampling models, which are essential for obtaining unbiased estimates of true abundance and population viability (Freckleton et al., 2006; Hobbs & Hooten, 2015).

However, there are many applications where incorporating observation and sampling models is not essential, and for which simpler models may provide useful insights. One such application, which is our focus here, is testing ecological hypotheses to explain changes in species abundance as a function of abiotic or biotic covariates. In this case, it is not necessary to know the true population abundance or the observation error, as long as the observation errors are homogeneous, or nearly so. Most importantly, the observation error cannot be correlated strongly with a predictor variable of interest. For example, if one wishes to test whether individual counts through time are a function of temperature, temperature must not strongly influence detection. If this assumption can be met, then a simple model structure may yield useful insights. This is fortunate because, as mentioned above, many existing population time‐series datasets lack replicates or other auxiliary data with which to properly fit observation models (e.g., repeat sampling, multiple observers, or mark–recapture data), but nevertheless contain information potentially useful for testing hypothesized drivers of population dynamics. The number of such datasets has greatly increased in recent decades (Comte et al., 2021; Dornelas et al., 2018).

Most population time series have some degree of temporal autocorrelation, meaning that the abundance at any point in time is dependent on one or more previous time steps (Barker & Sauer, 1992; Tuljapurkar & Haridas, 2006). This presents a challenge for testing hypotheses to explain abundance through time because abundance may be high despite unfavorable environmental conditions if it was even higher in a previous time step, or low despite favorable environmental conditions if it was even lower in a previous time step. Conversely, negative density dependence can cause populations to decline when abundances are high or increase when abundances are low, regardless of any environmental influence. Addressing these nuisances may be necessary for testing hypothesized drivers of population change.

We explore a range of regression models that differ mainly in how they account for temporal autocorrelation. At one end of the spectrum is a traditional generalized linear modeling (GLM) approach in which abundance at every time step is assumed to be independent of previous time steps. This simple model would likely be most suitable for highly fecund, short‐lived species (i.e., r life‐history strategists) whose populations undergo large fluctuations with low temporal autocorrelation. We refer to this as model A. At the other end of the spectrum, model C uses the difference in abundance between time steps (which can be interpreted as the population growth rate) as the response variable, an approach more appropriate for long‐lived species with low fecundity (i.e., K life‐history strategists) where populations change relatively slowly through time (i.e., their population time series have high temporal autocorrelation). This model can also readily accommodate density dependence. An intermediate model (model B) is a GLM with the same structure as model A, but with random effects modeled as temporally autoregressive. Model B represents abundance as a function of environmental covariates, as in Model A, but removes the assumption that successive counts are independent.

We describe the three models (Section 2) and evaluate them using simulated population data to test how they perform for species with different life‐history characteristics (Section 3). We then apply the models to two case studies using empirical data (Sections 4 and 5). The first case study uses a freshwater fish dataset to test hypotheses of associations between river flow conditions and abundance over time, and the second case study uses a small mammal dataset to test hypotheses of population response to precipitation and fire regimes. Finally, we discuss the results and provide recommendations based on the model comparisons (Section 6). Throughout, our perspective is pragmatic rather than theoretical: we wish to identify models that are useful for testing hypotheses to understand the change in abundance through time. Our hope is that this study provides useful guidance to ecologists and managers who are interested in testing hypotheses using existing time‐series datasets, particularly those datasets that lack information for fitting more complicated models.

## 2 THREE REGRESSION MODELS AND VARIANTS FOR TESTING HYPOTHESES TO EXPLAIN VARIATION IN ABUNDANCE OVER TIME

### 2.1 Model A. Generalized linear mixed model of abundance

For all models, we assume a dataset of counts comprised of individuals (*N*_*s*,*t*_) at one or more sites (*s*) at two or more points in time (*t*), with at least one candidate covariate (*X*_1*s*,*t*_) to explain variation in counts in space and/or time (in this section, we index the covariate by space and time, but in our examples, it is indexed only by time, with one exception for example 2). Because we are modeling count data, we use a generalized linear mixed model (GLMM) in which stochasticity is treated as conditionally Poisson (potentially with overdispersion) or negative binomially distributed. The simplest model, which we call “model A,” assumes no latent temporal autocorrelation in abundance after accounting for fixed effects.

(1) $$N_{s,t}\sim \text{Poisson}\left(\mu_{s,t}\right);\log\left(\mu_{s,t}\right)=\beta_{0}+\beta_{1}X_{1s,t}+\varepsilon_{s,t};\varepsilon_{s,t}\sim \text{Normal}\left(0,\sigma^{2}\right).$$

This is an overdispersed Poisson GLMM. If there are multiple sites, latent random effects $\varepsilon_{s,t}$ may not be fully independent, as sites may differ in mean abundances even after accounting for covariates. Therefore, a random intercept for site identity will usually be necessary. Random slopes for covariates may also be considered. This model is very similar to the basic Bayesian model for time‐series analysis at multiple sites presented by Kéry and Schaub (2012), which has been widely applied in ecological analyses. It can be fitted with common non‐Bayesian statistical packages or by Bayesian methods.

### 2.2 Model B. Generalized linear mixed model of abundance with autoregressive errors

Model B accounts for latent autoregressive dependence via the parameter *ρ* in formula 2 below. Most commonly, the autoregressive dependence is assumed to be Markov, i.e., dependent only on the previous time step (also called AR1 or a moving average model), although different dependence structures are possible.

(2) $$N_{s,t}\sim \text{Poisson}\left(\mu_{s,t}\right);\log\left(\mu_{s,t}\right)=\beta_{0}+\beta_{1}X_{1s,t}+\varepsilon_{s,t};\varepsilon_{s,t}\sim N\left(\rho \left(\varepsilon_{s,t-1}\right),\sigma^{2}\right).$$

This can be coded with relative ease in Bayesian software such as JAGS (Plummer, 2003) or stan (Stan Development Team, 2020), or it can be fit with the R packages glmmTMB (Brooks et al., 2017) and brms (Bürkner, 2017). One consequence of adding the autoregressive term is that this model requires more temporally complete data than model A because at least two consecutive time steps of data are required to estimate the AR1 error term. However, missing count data can be imputed if Bayesian methods are used or a custom model is written. The model implicitly assumes that time steps are equal.

### 2.3 Model C. Generalized linear model of growth rate

Model C (Equation 3) characterizes the change in abundance through time—i.e., the population growth rate.

(3) $$N_{s,t}\sim \text{Poisson}\left(\mu_{s,t}\right);\log\;\left(\mu_{s,t}\right)=\log\left(\mu_{s,t-1}\right)+\beta_{0}+\beta_{1}X_{1s,t}+\varepsilon_{s,t};\varepsilon_{s,t}\sim N\left(0,\sigma^{2}\right).$$

This model cannot be fit in this form using conventional non‐Bayesian regression packages (although see Equation 5 for a reformulation), but can be estimated with Bayesian methods. We can include an autoregressive abundance term (on the original scale, not logged) to serve as a density dependence term (Hobbs & Hooten, 2015; Equation 4).

(4) $$N_{s,t}\sim \text{Poisson}\left(\mu_{s,t}\right);\log\;\left(\mu_{s,t}\right)=\log\left(\mu_{s,t-1}\right)+\beta_{0}+\beta_{1}X_{1s,t}+\beta_{2}\mu_{s,t-1}+\varepsilon_{s,t};\varepsilon_{s,t}\sim N\left(0,\sigma^{2}\right).$$

Coefficient *β*_2_ will usually take on a negative value, representing negative density dependence, i.e., the reduction in growth rate as N increases. The intercept in this model (*β*_0_) can now be interpreted as the intrinsic growth rate or the growth rate when *N* is close to 0. This model formulation is a form of the Ricker model, with carrying capacity calculated as $K=\frac{\beta_{0}}{-\beta_{2}}$, after accounting for exogenous influences on population change ($\beta_{1}X_{1s,t}$). For simplicity, we use Equation 3 for model C in the simulations and examples in this study, but we return to the topic of density dependence in Section 6.

Like model B, model C implicitly assumes that time steps are approximately equal, and is not appropriate for datasets with many missing data points unless using Bayesian methods or a custom model that allows for imputation. Unlike models A and B, a random effect for site identity may not be necessary because differences in mean abundances among sites are accounted for by modeling changes in abundance rather than absolute abundance. However, if some sites have higher or lower long‐term growth rates than can be explained by covariates, random effects may be required. Random slopes may also be considered if covariates affect the growth rate differently due to context‐dependent processes that vary in space.

Although this model cannot be fit using conventional frequentist statistical packages, an approximation using conventional frequentist regression methods is possible if the dataset contains no zeros, or if 1 is added to all counts to preclude non‐zero values. We rearrange the model to bring the lagged abundance term to the left side and omit the Poisson stochasticity (Equation 5).

(5) $$\;\log\left(N_{s,t}/N_{s,t-1}\right)=\beta_{0}+\beta_{1}X_{1s,t}+\varepsilon_{s,t};\varepsilon_{s,t}\sim N\left(0,\sigma^{2}\right).$$

In the previously presented models, the data used are the abundances, but here the data are the log of the ratio of abundance in time *t* to abundance in time *t* − 1, which is equivalent to log(*N*_s*,t*_) − log(*N*_*s*,*t*−1_). This makes it possible to fit the model using any linear regression software. Models of similar form have been used in ecological time‐series analysis previously, although often with a coupled observation model (e.g., Williams et al., 2003).

For all three models, observation error is a problem if it is correlated with one or more variables of interest, as mentioned previously. If a variable (say, temperature) affects both abundance and detection, the individual effects cannot be separated using the models discussed here. However, if detection varies predictably with an environmental variable that is *not* of research interest and is not strongly correlated with a variable of interest, then it may be possible to include a covariate to account for the observation bias and obtain an unbiased estimate of the covariate that is of interest. This does not require a separate observation model; the covariate can be simply included as a linear term (Barker et al., 2018). We illustrate this idea in the case study presented in Section 4.

## 3 SIMULATIONS

**FIGURE 1:**
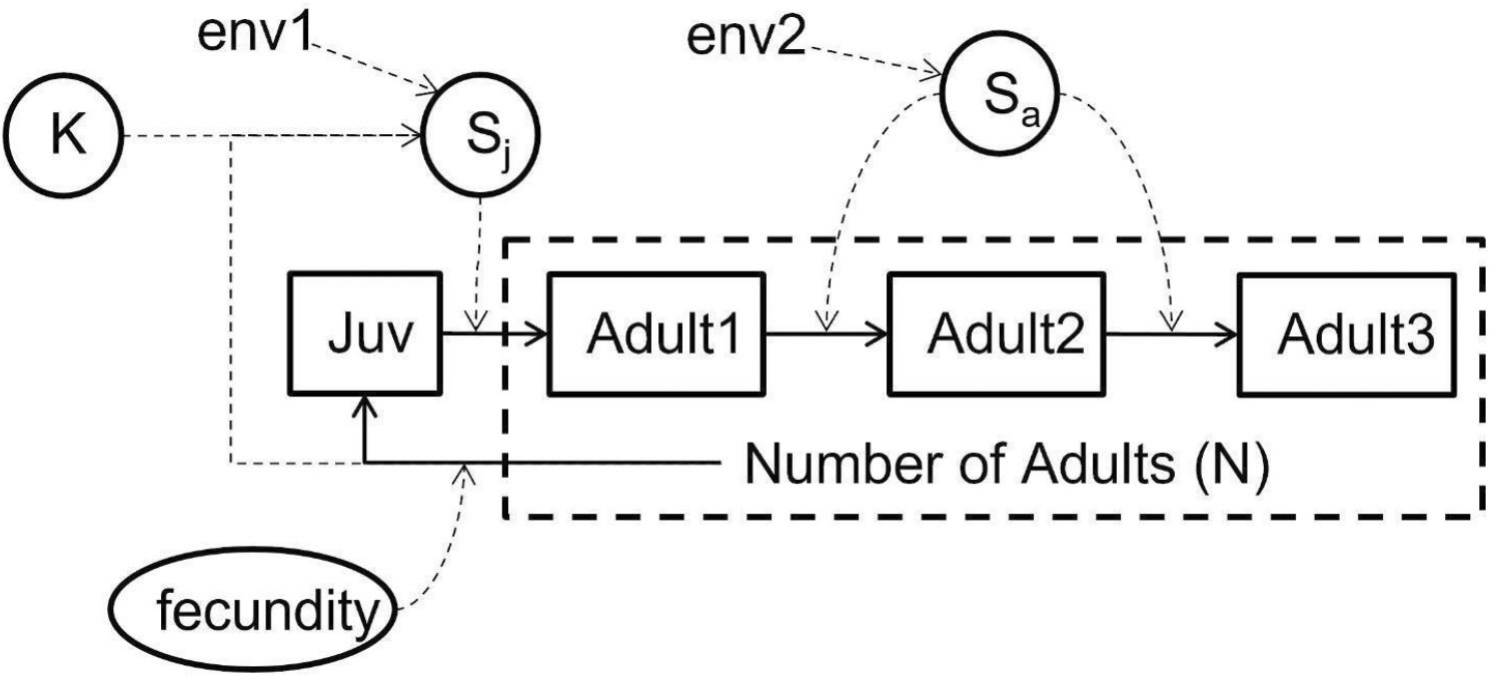
Structure of simulation model. Solid arrows indicate transitions; dashed arrows indicate influence. See text for details. This shows one juvenile stage and three adult stages as an example, but the number of stages can be user defined.

We simulated population time series to compare the performance of the Bayesian and non‐Bayesian versions of the three models when fitted to datasets representing populations that varied by fecundity and survival. Our simulations were based on a matrix modeling approach that accounted for life span, reproductive age, juvenile survival rate, adult survival rate, fecundity (technically, this is fecundity multiplied by egg‐to‐juvenile survival), carrying capacity, initial population size, and length of simulation (Figure 1).

We assumed that juvenile survival (*S*_*j*_) was affected by a single time‐varying environmental variable (*env1*), adult survival (*S*_*a*_) was affected by a different time‐varying environmental variable (*env2*), and that *S*_*j*_ and *S*_*a*_ did not covary. These environmental variables were drawn from normal distributions (one draw per time step, *t*) with a mean of zero and were added to *S*_*j*_ and *S*_*a*_ on the logit scale, after which the variables were back‐transformed to probabilities. Survival was calculated independently for each year class as a binomial process based on the applicable (juvenile or adult) survival probability. Recruitment was modeled as a Poisson process based on the number of reproductive adults and fecundity. Density dependence was incorporated into the juvenile survival term by multiplying *S*_*j*_ by 1 − (*N*_*t*−1_/*K*), where *K* is the carrying capacity. All code is provided with the data in the Zenodo archive.

In our first round of simulations, we ran a large number of iterations representing two scenarios. Scenario 1 specified high fecundity (4 juveniles per adult), low survival (*S*_*j*_ = 0.25, *S*_*a*_ = 0.4), a carrying capacity of 1000, and a lifespan of 3 years. Scenario 2 specified low fecundity (0.5 juveniles per adult), high survival (*S*_*j*_ = 0.5, *S*_*a*_ = 0.9), a carrying capacity of 500, and a lifespan of 12 years. To test our models, we summed the adult population at each time step and modeled N as a function of env1 and env2 using each of the three model structures. We predicted that models A and B would be favored for Scenario 1 (a population of a high‐fecundity, short‐lived species with low temporal autocorrelation), and that model C would be favored for Scenario 2 (a population of a low‐fecundity, long‐lived species with high temporal autocorrelation), with model B in second place. For the Bayesian models, we ran 1000 simulations of 50 years each for both scenarios using RunJAGS (Denwood, 2016) and JAGS (Plummer, 2003). For each simulation, we ran four chains for a burn‐in period that varied by the model (5000 for model A; 20,000 for model B; and 10,000 for model C, based on tests to ensure convergence) followed by a 20,000‐iteration sampling period. For the non‐Bayesian models, we ran 1000 simulations of 50 years each for both scenarios using glmmTMB (Brooks et al., 2017). We considered numerous indicators of model performance but determined that most were inappropriate for comparison between Bayesian and non‐Bayesian models that have different response variables (e.g., likelihood‐based information criteria such as AIC or BIC are not an option). We elected to compare models based on the squared correlation between model predictions and actual values (a pseudo‐*R* ^2^), a simple but admittedly imperfect metric because it does not consider model complexity or out‐of‐sample performance. In all cases, model predictions only included fixed effects, not error terms.

We found that models A and B had better average performance than model C for scenario 1, consistent with predictions (Table 1). Nevertheless, model C was the best‐supported model for about a quarter of the datasets. Bayesian and non‐Bayesian models had nearly identical average performance, but for non‐Bayesian models, model B had a performance that was consistently just slightly lower than model A, and therefore was rarely selected as best overall. However, the actual performance difference between models A and B was very small, so we caution against interpreting model B as inferior. Model A has the benefit of simplicity, while model B is arguably a “safer” choice because it does not assume independence between years, and therefore does not risk underestimating the standard errors of parameter estimates. For scenario 2 we found that model C consistently had the highest pseudo‐*R* ^2^. Models A and B had very similar performance to each other, but much lower performance than model C.

**TABLE 1:** Predictions and results of simulations of the three models under two scenarios. The “% each model selected as best” indicates the frequency with which each model had the lowest mean absolute percent error (MAPE) in 1000 random model runs. The “pseudo‐*R* ^2^” is the squared correlation between model predictions and actual values.

Scenario	Scenario characteristics	Prediction	Model type	% of each model selected as best			Pseudo‐*R* ^2^		
				A	B	C	A	B	C
1	High fecundity, short‐lived (*r*‐strategist)	A, B, and C similar	Non‐Bayesian	69%	6%	23%	.44	.42	.35
			Bayesian	49%	26%	25%	.42	.41	.35
2	Low fecundity, long‐ lived (*k*‐strategist)	C over B over A	Non‐Bayesian	0%	0%	100%	.20	.19	.67
			Bayesian	0%	0%	100%	.19	.19	.67

We ran a second round of simulations in which we compared models based on predictor variables that were strongly correlated with abundance (i.e., env1 and env2, as used in the first round of simulations) with a weakly correlated predictor variable (env3) that was based on the sum of env1 and env2 but had substantial random noise added. For this comparison, we only used the three non‐Bayesian models and evaluated them based on their ability to predict abundance in the final year (i.e., the mean absolute percent error for year 50), which was withheld from model fitting. This also provided an out‐of‐sample estimate of model error. We ran 1000 iterations for each of the same two scenarios used in the first round. We hypothesized that the “strong” model would be selected the great majority of the time in all cases. However, our results indicated that the “weak” model was selected almost half the time in some cases, and at least a third of the time in all cases (Table 2). This was true in spite of the fact that mean error rates were higher for the “weak” models in both scenarios. This serves as a reminder to be cautious in interpreting the results of an analysis with a single time series—even a 49‐year time series—as providing strong evidence in favor of one hypothesis over another. By random chance, a weak variable can have a tighter correlation with abundance than a strong variable in any individual dataset. Unless random variability is quite low, multiple datasets are needed to have high confidence in the outcomes of a test of two competing, correlated predictor variables.

**TABLE 2:** Results of simulations to test a “strong” model (with predictors directly correlated with abundance) versus a “weak” model (with predictors indirectly correlated with abundance and added noise). The “% of times the ‘strong’ model selected as best” indicates the frequency with which the strong model had lower mean absolute percent error (MAPE) than the weak model, in 1000 random model runs. The “mean error rate” indicates the average MAPE for the strong and weak models in each scenario.

Scenario	Scenario characteristics	% of times the “strong” model selected as best			Mean error rate for “strong”/“weak” model		
		A	B	C	A	B	C
1	High fecundity, short lived (*r*‐strategist)	64%	62%	55%	58/78%	49/63%	66/74%
2	Low fecundity, long lived (*k*‐strategist)	56%	59%	65%	30/32%	27/28%	7/10%

Across our simulations, we found that all three models produced similar parameter estimates in most cases, although model A tended to have lower standard errors on parameters than models B and C. We consider the standard errors of model A to be biased low since this model assumes independence of annual samples, an assumption we know is not met in Scenario 2. In these simulations, we cannot evaluate the accuracy of parameter estimates against known values because the simulation model is substantially more complicated than any of the three fitting models. Nevertheless, we have found these simulations to be a useful tool for evaluating model behavior and performance. The simulations we report here are just a few of those that are possible, and we encourage users to adapt the supplied simulation code to conduct other comparisons relevant to particular applications.

## 4 CASE STUDY 1. FLOW ECOLOGY OF SHOAL FISHES IN THE ETOWAH RIVER, GEORGIA, UNITED STATES

Aquatic organisms that have evolved in riverine ecosystems are assumed to be adapted to a flow regime (Lytle & Poff, 2004; Poff et al., 1997). Although researchers have demonstrated that fish communities are structured by patterns in natural (Mims & Olden, 2012; Poff & Allan, 1995) and altered flow regimes (Bunn & Arthington, 2002; Kiernan & Moyle, 2012; Perkin & Bonner, 2011), species‐specific or trait‐specific models of organismal response to interannual variability in flows are still largely lacking (Freeman et al., 2022). It has been suggested that these flow ecology questions could be better answered with the use of population *rates* (e.g., growth rate) rather than *states* (e.g., abundance) as response variables in time‐series data analyses (Poff, 2018; Tonkin et al., 2019; Wheeler et al., 2018). Model C can be viewed as a rate model, whereas models A and B are repeated‐state models (i.e., models of abundances observed repeatedly through time).

We used a long‐term dataset of fish counts from the Etowah River in Georgia, USA (Freeman et al., 2017), a tributary of the Coosa River that supports a diverse fish assemblage, including several imperiled species of conservation interest. Fish were collected using seines annually in the fall (September–October) at 10 sites between 1997 and 2016, although collections were not made in 2010 and 2011, and some sites were not sampled in some years due to persistently high‐flow conditions that made sampling unsafe and ineffective. For this analysis, we focused on six small‐bodied shoal‐dwelling fish species, all considered *r*‐strategists, which generally mature at 1 year of age and are known to spawn in the late spring and early summer (Table 3). We standardized abundance over different sampling efforts for each site by dividing the number of individuals of each species by the number of samples conducted at each collection event and multiplying by 100.

**TABLE 3:** Parameter estimates (posterior means and standard deviations) and performance scores for the Bayesian versions of the three model types for six fish species. “High flow” and “Low flow” are variables representing the number of high‐flow days and low‐flow days in the current year. “lag” indicates the same variable for the prior year. “*Q*” is the discharge on the day of sampling. Superscripts indicate support for hypotheses of the corresponding number (i.e., parameter estimates with the expected sign and 90% credible intervals that do not overlap zero). *R* ^2^ is the squared correlation between conditional model predictions and observations (a pseudo‐*R* ^2^).

Species	Model	High flow	High flow lag	Low flow	Low flow lag	*Q*	DIC	*R* ^2^
*Cyprinella callistia* Alabama shiner	A	−0.49 (0.09)^1^	−0.11 (0.06)	−0.01 (0.07)	0.01 (0.07)	−0.42 (0.07)	1207	.39
	B	−0.45 (0.09)^1^	−0.07 (0.06)	0.03 (0.07)	0.02 (0.06)	−0.43 (0.07)	1207	.37
	C	−0.19 (0.08)^1^	0.30 (0.07)^2^	0.28 (0.08)	−0.30 (0.07)^4^	−0.25 (0.07)	1210	.54
*Macrhybopsis etnieri* Coosa chub	A	−0.13 (0.11)	−0.01 (0.07)	−0.04 (0.08)	−0.12 (0.08)	−0.05 (0.09)	1085	.05
	B	−0.13 (0.11)	−0.01 (0.07)	−0.04 (0.09)	−0.13 (0.08)	−0.04 (0.09)	1085	.04
	C	0.07 (0.12)	0.17 (0.10)	0.17 (0.12)	−0.13 (0.11)	−0.02 (0.11)	1088	.04
*Noturus leptacanthus* Speckled madtom	A	−0.70 (0.16)^1^	−0.08 (0.08)	−0.26 (0.10)^3^	0.02 (0.09)	−0.25 (0.11)	706	.10
	B	−0.69 (0.16)^1^	−0.08 (0.08)	−0.25 (0.10)^3^	0.02 (0.09)	−0.25 (0.11)	707	.07
	C	−0.48 (0.16)^1^	0.63 (0.15)^2^	−0.04 (0.13)	0.00 (0.12)	−0.15 (0.13)	720	.30
*Noturus* sp. cf. *munitis* Coosa madtom	A	−0.28 (0.14)^1^	−0.18 (0.09)	0.10 (0.11)	−0.20 (0.10)^4^	0.12 (0.11)	821	.06
	B	−0.23 (0.14)	−0.14 (0.09)	0.13 (0.10)	−0.18 (0.10)	0.15 (0.11)	820	.03
	C	−0.07 (0.14)	0.04 (0.13)	0.36 (0.13)	−0.39 (0.12)^4^	0.38 (0.13)	821	.17
*Percina nigrofasciata* Blackbanded darter	A	−0.55 (0.10)^1^	−0.24 (0.06)	−0.17 (0.07)^3^	−0.15 (0.07)^4^	−0.45 (0.08)	1076	.30
	B	−0.53 (0.10)^1^	−0.23 (0.06)	−0.18 (0.07)^3^	−0.15 (0.07)^4^	−0.45 (0.08)	1078	.35
	C	−0.28 (0.11)^1^	0.47 (0.09)^2^	−0.14 (0.09)	−0.09 (0.09)	−0.45 (0.09)	1084	.36
*Percina palmaris* Bronze darter	A	−0.48 (0.11)^1^	0.04 (0.06)	−0.03 (0.08)	−0.15 (0.07)^4^	−0.43 (0.08)	1064	.22
	B	−0.40 (0.11)^1^	0.05 (0.06)	0.02 (0.07)	−0.15 (0.07)^4^	−0.40 (0.08)	1064	.21
	C	−0.18 (0.10)^1^	0.64 (0.08)^2^	0.22 (0.09)	−0.29 (0.08)^4^	−0.27 (0.09)	1065	.41

We proposed four hypotheses regarding how the abundances of fish species respond to flow:

1. Populations decline in years of exceptionally high summer flows due to direct mortality of eggs and young of the year, which reduces total abundance (Harvey, 1987; Humphries et al., 1999).
2. Populations increase in years following exceptionally high summer flows due to the scouring of fine sediment that increases the productivity of the system in the following year (Cattanéo et al., 2001). Alternatively, populations could rise due to a density‐dependent response to declines, or to observation/sampling error that mimics density dependence (Freckleton et al., 2006); it may not be possible to disentangle these mechanisms.
3. Populations decline in years of exceptionally low summer flows due to reduced habitat volume and productivity, although this effect could be masked if individuals immigrate to sampled sites from adjacent, less suitable habitats (Falke et al., 2010; Hakala & Hartman, 2004; Hedden & Gido, 2020).
4. Populations also decline in years following exceptionally low flows (Rolls et al., 2012).

We calculated high‐ and low‐flow metrics for the Etowah River from the USGS gage at Canton, GA (gage 02392000; U.S. Geological Survey, 2021), based on the 90th percentile and 10th percentile daily flows for the period of record (1896–present except for the years 1906–1935). For every year for which we had fish data, we calculated the number of days above the 90th percentile (high‐flow days, HFD_*t*_) and number of days below the 10th percentile (low‐flow days, LFD_*t*_) for the summer, which we defined as June through September. We used the same flow metric values for each site within a year (i.e., flow metrics vary by time but not space).

For each species, we fit the Bayesian versions of models A–C using RunJAGS (Denwood, 2016) and JAGS (Plummer, 2003). We tested all hypotheses simultaneously by including fixed effects for HFD_*t*_, HFD_*t*−1_, LFD_*t*_, and LFD_*t*−1_. We also included a term for mean discharge on the day of sampling (*Q*), hypothesizing that capture efficiency would be negatively related to stage height (this term was only moderately correlated with other flow variables; Pearson's *r* < .5). Correlations among other predictor variables also were low (Pearson *r* = .42 or less). For consistency, we included a random intercept for the site in all models. All predictor variables were standardized by subtracting the mean and dividing by the standard deviation. We specified vague priors for all parameters. We ran four chains for a 30,000‐iteration burn‐in period (including a 2000‐iteration adaptation) followed by a 100,000‐iteration sampling period with a thinning factor of 10, for a total of 40,000 samples included in the posterior parameter estimates. We determined convergence based on the Brooks–Gelman–Rubin diagnostic (R‐hat <1.1). When necessary, model runs were extended to achieve convergence. We calculated the deviance information criterion (DIC) as an indicator of the relative support for each set of three models for each species. We also calculated a pseudo‐*R* ^2^ as the squared correlation between marginal (i.e., without random effects) model predictions and observations. Code is provided with the data in the Zenodo archive.

All algorithms converged. We found that, based on DIC, models A and B had very similar performance; the slightly better likelihood of model B was balanced by its slightly greater complexity (Table 3). Model C had similar support to models A and B for most species, although it had substantially lower support for the speckled madtom and the blackbanded darter. Based on pseudo‐*R* ^2^, model C had higher support than models A and B for all species except the Coosa chub.

We found mixed support for our hypotheses (Table 3). Hypothesis 1 (negative effect of high flows) was generally supported, with negative parameter estimates for almost all models for all species. We found support for hypothesis 2 (positive effect of lagged high flows) for four species, but only with model C. Hypothesis 3 (negative effect of low flows) was generally not supported, with parameter estimates ranging from weakly negative to weakly positive across species and models, with the exception of the speckled madtom and the blackbanded darter. This could indicate a lack of an effect or that negative effects of low flows on abundances were masked by aggregation of individuals (Falke et al., 2010; Hakala & Hartman, 2004; Hedden & Gido, 2020). Hypothesis 4 (negative effect of lagged low flows) was moderately supported, with generally negative parameter estimates, although these were imprecisely estimated for half of the species. Support for Hypothesis 4 was generally more evident with model C than with other models. All species except the Coosa madtom had the expected negative parameter estimate for discharge on the day of sampling. Broadly speaking, parameter estimates were quite similar between models A and B for a given species, but model C tended to have parameter estimates that differed from the other two models. We explore this in Section 6.

We also ran non‐Bayesian versions of all models for all species. The results, reported in the Supporting Information (Table SI1), were very similar to the Bayesian model results in most cases, although the standard errors on parameter estimates tended to be substantially smaller for model A.

## 5 CASE STUDY 2. SMALL MAMMALS IN KONZA PRAIRIE, KANSAS, UNITED STATES

Temporal changes in the community composition of small mammals have been linked to interannual climate variation, especially precipitation (Bruckerhoff et al., 2020; Cárdenas et al., 2021; Thibault et al., 2010). Mammal species representing different feeding guilds are hypothesized to respond differently to variations in precipitation given the distinct effects of rainfall on different mammal food resources (Reed et al., 2006b), but these predictions have not been widely tested. We assessed whether populations of small mammal species at Konza Prairie Biological Station (KPBS) respond differentially to precipitation based on their feeding guilds. We also assessed the role of the burning regime as burning, and grazing treatments are applied at the watershed scale at KPBS, a tallgrass prairie preserve, and Long‐Term Ecological Research site in the Flint Hills of Northeastern Kansas, USA.

Small mammal data from KPBS consisted of annual autumn sampling from 1992 to 2012 in six watersheds. The upland and lowland portions of each watershed were surveyed with 20 stations of two Sherman live traps. We used annual total precipitation at KPBS as our climatic variable of interest. We also examined the role of time since the last burn; prescribed burns have been carried out at intervals of 1, 4, or 20 years depending on the watershed. Correlations among predictor variables were low (Pearson *r* < .01). We included the six most common small mammal species in our analysis (Table 4) and classified the feeding guild of each species based on Reed et al. (2006b).

**TABLE 4:** Parameter estimates (posterior means and standard deviations) and performance scores for the Bayesian versions of the three model types for six mammal species. “Precipitation” and “Time since burning” are variables representing the amount of precipitation in the preceding year and the number of years since prescribed burns occurred at the site. Superscripts indicate support for hypotheses of the corresponding number (i.e., parameter estimates with the expected sign and 90% credible intervals that do not overlap zero). *R* ^2^ is the squared correlation between conditional model predictions and observations (a pseudo‐*R* ^2^).

Species	Model	Precipitation	Time since burning	DIC	*R* ^2^
*Microtus ochrogaster* Prairie vole (herbivore)	A	0.76 (0.26)^1^	0.57 (0.28)	301.0	.29
	B	0.66 (0.28)^1^	0.56 (0.32)	297.9	.29
	C	0.32 (0.34)	0.14 (0.25)	297.6	.02
*Sigmodon hispidus* Hispid cotton rat (herbivore)	A	−0.16 (0.28)	0.44 (0.35)	282.3	.06
	B	−0.22 (0.28)	0.33 (0.39)	283.8	.05
	C	−0.21 (0.30)	−0.05 (0.25)	282.7	.01
*Blarina hylophaga* Elliot's short‐tailed shrew (insectivore)	A	0.49 (0.17)^2^	0.14 (0.19)	364.5	.03
	B	0.46 (0.18)^2^	0.13 (0.19)	365.7	.04
	C	0.85 (0.20)^2^	−0.05 (0.14)	373.9	.12
*Peromyscus leucopus* White‐footed mouse (omnivore)	A	−0.07 (0.10)	0.44 (0.14)	526.6	.58
	B	−0.08 (0.10)	0.42 (0.17)	525.6	.58
	C	−0.01 (0.11)	0.04 (0.08)	529.3	.00
*Peromyscus maniculatus* Deer mouse (omnivore)	A	0.06 (0.07)	−0.41 (0.14)^4^	586.8	.27
	B	0.04 (0.07)	−0.44 (0.15)^4^	587.4	.28
	C	0.00 (0.08)	−0.06 (0.08)	594.6	.00
*Reithrodontomys megalotis* Western harvest mouse (granivore)	A	0.20 (0.13)	−0.18 (0.19)	438.1	.00
	B	0.27 (0.14)	−0.10 (0.20)	437.1	.00
	C	−0.01 (0.23)	−0.09 (0.21)	442.4	.00

We proposed four hypotheses about how small mammal populations would fluctuate with precipitation and burning regime:

1. Populations of herbivores increase in years with higher precipitation, as more rainfall generates increased forage biomass (Reed et al., 2006a, 2007).
2. Populations of insectivores and omnivores also increase in high rainfall years, as insect food sources are positively associated with increases in plant biomass (Kaufman et al., 2012; Reed et al., 2006a).
3. Granivore populations decline in years with high precipitation because increased plant litter negatively affects seed predation (Reed et al., 2006b).
4. Increased time since burning has an overall negative effect on populations across long time periods. While the relationship between woody cover and the abundance of several species is not monotonic, the lowest levels of abundance and small mammal richness occur at the highest levels of forest cover, i.e., the greatest time since burning (Matlack et al., 2008).

We used Bayesian versions of models A, B, and C to analyze the mammal dataset. Each model included as predictors annual precipitation and time since burning—each scaled by subtracting the mean and dividing by the standard deviation—along with a random effect for the site. Precipitation varied in time but not among sites, whereas time since burning varied in both dimensions. Models were fit and compared using the same methodology and specifications as Example 1, albeit with a shorter burn‐in period (20,000 iterations). Code is provided with the data in the Zenodo archive.

All algorithms converged. The top‐ranked model formulation varied among taxa (Table 4). As with the fish analysis, models A and B diverged little in model fit for five of six species based on DIC (i.e., ΔDIC ≤2). These two models performed better than model C (i.e., ΔDIC >2) for four species (Eliot's short‐tailed shrew, white‐footed mouse, deer mouse, and Western harvest mouse), whereas models B and C performed similarly and were the top‐ranked models for prairie vole. All three models performed similarly in the case of the hispid cotton rat. Pseudo‐*R* ^2^ based strictly on fixed effects varied considerably among taxa, and also generally indicated greater support for models A and B.

We found mixed support for our hypotheses (Table 4). Annual precipitation was associated with increases in counts of one of the two herbivores (prairie vole; Hypothesis 1) as well as the insectivore (Eliot's short‐tailed shrew; Hypothesis 2), but not for the two omnivores (white‐footed mouse and deer mouse; Hypothesis 2), where the effect of precipitation did not differ from zero based on Bayesian credible intervals (BCI). The lack of a strong rainfall effect in deer mouse was consistent with a short‐term demographic analysis of this taxon at KPBS, in which the highest population growth rate occurred during a year of average precipitation, as opposed to a very wet or dry year (Reed et al., 2007). We expected the granivorous Western harvest mouse to decline with precipitation (Hypothesis 3) but this species also showed a small positive association with rainfall in the top models. Species had mixed responses to time since burning, with only deer mouse exhibiting the hypothesized negative response in the top‐ranked models compared to two species with positive responses based on BCI (prairie vole and white‐footed mouse). Parameter estimates for the predictors of interest in models A and B were similar: they had the same sign (i.e., positive, negative, or zero according to BCI) in 11 of 12 cases, and differed in magnitude by <25% in most cases. Parameter estimates from model C, however, deviated in several instances from those of A and B, particularly for the time‐since‐burning covariate. Results of the non‐Bayesian models (Table SI2) were qualitatively similar to the Bayesian model results in most cases (e.g., most parameter estimates have the same sign and similar magnitude). As for Case Study 1, we found that model A had much smaller standard errors on parameter estimates than models B and C.

## 6 DISCUSSION

Time‐series datasets of species abundances have become more widely available in recent decades (Comte et al., 2021; Dornelas et al., 2018), offering increasing opportunities to test hypotheses to explain variation in species abundances through time. Our objective was to evaluate the potential for simple statistical models to test such hypotheses. We found that even the simplest model (model A) was useful for detecting relationships between predictors and abundances, particularly for *r*‐strategists (shorter‐lived and higher fecundity species), although a model with autoregressive errors (model B) and a model of growth rate (model C) was sometimes preferred in both simulations and case studies. In simulations of time series of *K*‐strategists (longer‐lived and lower fecundity species), models B and C were usually preferred. The species in both of our case studies were closer to *r*‐strategists than *K*‐strategists, but we nevertheless found that model C was preferred for several species. Based on our results, our general recommendation is to test all three models, rather than trying to determine a priori which is likely to be the best supported based on species traits or characteristics of the time series. After the data are formatted, all three models are straightforward to implement. Of course, depending on the research question, there could be reasons to select one model over another (e.g., if questions concern population growth rate, model C would be preferred).

The fact that model C revealed relationships not evident in models A and B is an important result and is a further rationale to fit multiple models. Such discrepancies among models can provide insight into the mechanisms giving rise to observed population patterns, and (potentially) evidence in favor of one or more alternative hypotheses (Yen et al., 2021). Case Study 1 provides an illustration: evidence for hypothesis 2 (populations increase in years following exceptionally high flows) is only provided by model C. Models A and B do not reveal this relationship because they use abundance rather than growth rate as a response variable, and abundances tend to be low in years following exceptionally high flows because populations are still recovering from the even lower levels in the preceding year. The fact that populations tend to rebound in the year after the high flow is only evident when using the growth rate as the response variable (similar patterns of lagged high‐flow effects are well documented in the fish ecology literature; Gido et al., 2013; Humphries et al., 2008; Rogosch et al., 2019). Because the models assess different things, we advise fitting multiple models and interpreting outputs from each.

Model C has one advantage over the other models: because it measures change, abundance is entirely factored out of the regression equation. This implies the model can be used to simultaneously analyze multiple datasets collected with different sampling methods, as long as such methods have been used consistently within each dataset. Of course, the same predictor variables must be available in each dataset to make such comparisons, and the assumption of homogeneity of observation error still applies. Setting up such a multi‐dataset model is straightforward with the non‐Bayesian version of model C, although it requires more work with the Bayesian version.

On the other hand, model C will not be as useful for datasets that (1) lack sufficient year‐to‐year variability or (2) have spatial variability rather than temporal variability in relationships of interest. The first case is perhaps best illustrated with an extreme example: consider a hypothetical 10‐year dataset in which the environmental conditions are poor for 5 years running and then good for 5 years running. Further, assume that the population responds by staying at a steadily low level for 5 years and then at a steadily high level for 5 years. Because there is overall little information on the change in such a dataset, model C will perform poorly, whereas models A and B should perform quite well. This is a toy example, but the more general point is that to test hypotheses about the factors governing population increases and decreases, the dataset must have sufficient dynamics in both the population response and predictors. Three mammal taxa in case study 2 illustrate the second case in which model C may be inappropriate. For these species—prairie vole, white‐footed mouse, and deer mouse—sites with above‐average time‐since‐burning values had variable but typically higher species abundances (or lower in the case of deer mouse) than sites with more frequent burning. Models A and B identified this correlation between time since burning and overall abundance while model C did not because the greatest variability in both abundance and the predictor occurred across space rather than through time. Therefore, for datasets in which effects of interest vary spatially, model C may not be as useful as models A and B.

Datasets with many gaps present a problem for the non‐Bayesian versions of models B and C when using conventional regression packages. Fitting models B and C requires data from both the current and the prior time step, thus one missing value can effectively eliminate two observations from the time series (i.e., 2 years of data). Where sampling is conducted every other year or every few years, which is often employed in wildlife and fish population monitoring designs (Urquhart & Kincaid, 1999), model A becomes the only choice available if conventional frequentist methods are used. The Bayesian versions of the models allow imputation of missing data, although we have not tested their performance when frequency and intervals in gaps are large.

The simplicity of Model A is appealing, but outputs from this model must be interpreted carefully as the assumption of independence of errors is unlikely to be met in many time‐series datasets. We found that the precisions of the parameter estimates from non‐Bayesian model A were overly optimistic in both simulations and case studies. This did not appear to be an issue with the Bayesian version of model A, however.

We expect that some readers may be concerned with the lack of an observation model. Occupancy models (MacKenzie et al., 2002), N‐mixture models (Royle, 2004), and their variants have become standard methods for many ecologists and fisheries and wildlife practitioners, despite the additional data requirements and serious questions about their reliability under certain conditions (Barker et al., 2018; Link et al., 2018). Our objective is not to discourage the use of observation models for datasets with the information to parameterize them. Rather, we seek to encourage the use of accessible methods for analyzing long‐term datasets when the information needed for observation models is lacking (e.g., monitoring programs that were initiated prior to the widespread use of repeat sampling methods), or when the statistical programming and time required for implementing a hierarchical model with an observation component renders such methods impractical. As shown in Case Study 1, variables that affect detection can be included as covariates in the regression equation; we suspect that this can address detection bias as effectively as an observation model, or nearly so, in many cases (also see Barker et al., 2018). However, if the variables affecting detection are highly correlated with variables hypothesized to affect the abundance, then it is impossible to distinguish detection effects from abundance effects. In such cases, an observation model (and the additional data to parameterize such a model) is necessary.

All models presented herein are variants of linear regression, and their appropriateness for testing hypotheses depends on whether those hypotheses can be represented in the form of a linear relationship with the response. Reality is complex, and a hypothesis such as “populations of herbivores increase in years with higher precipitation, as more rainfall generates increased forage biomass” attempts to identify the most essential relationship in a cascade of influences and interactions involving spatially variable soils, timing of precipitation, the size of other herbivore populations, etc. If a hypothesis is not supported in the form that it is tested, it could be because a key aspect of the process is missing, or because the metric chosen does not have a linear relationship with the response. Sometimes relationships can be linearized via transformations (e.g., saturating relationships can be made more linear by log transformation of the predictor) or by including polynomial terms. In our examples, we did not compare alternative metrics for representing our hypothesis, but if we were conducting these analyses in earnest (rather than demonstrating methods), we would certainly consider other possibilities.

Density dependence is another potential nuisance. Although we do not incorporate density dependence into Model C in the simulations or examples reported here, in separate explorations we have frequently found support for including this parameter. In these tests, we have nearly always found that the parameter estimate was negative, indicating negative density dependence. Including a density dependence term has the additional advantage of accounting for observation and sampling error that mimics negative density dependence (Freckleton et al., 2006). By random variation, a particular observed count could be exceptionally high or low, but such anomalies are unlikely to be followed by a similarly extreme estimate in the next time step. The result is that populations will appear to increase rapidly after an unusual dip, and to decline after an unusual peak, a pattern similar to negative density‐dependent behavior. However, unless density dependence is the focus of the analysis, it is of little consequence whether the density dependence term represents true or apparent density dependence. The term serves the dual purpose of accounting for both, with a caveat that the unique contributions of each may not be identifiable.

The choice between non‐Bayesian and Bayesian methods is mostly a practical one. We have observed that the non‐Bayesian and Bayesian versions of all three models generally yield very similar parameter estimates. We find the non‐Bayesian model C to be somewhat unsatisfying in that it requires the ad hoc solution of adding 1 to counts of 0. Nevertheless, our simulations indicated that the non‐Bayesian version of model C can detect relationships between predictors and response where they are present, and so is likely to be sufficient for many purposes. One analytical strategy could be to first test models using the non‐Bayesian methods, and to invest the effort into the Bayesian models only if results are sufficiently interesting or the application is sufficiently important. This two‐step process may be favored when there are large numbers of hypotheses to be tested, as preliminary screening can be conducted much more quickly with the non‐Bayesian models.

Long‐term abundance datasets were once a scarce resource, but they have been quietly accumulating in recent decades. There are now many hundreds, likely thousands of such datasets, although many lack the auxiliary data necessary to parameterize a linked observation model. We were thus motivated to explore relatively simple, accessible methods that could be used to make valid inferences from time‐series datasets without an observation model. Despite limitations potentially imposed by unknown observation biases, we believe that unlocking the information in these datasets could contribute greatly to ecological understanding. We hope that our results will encourage others to use the models presented here as starting points to investigate environmental effects on population dynamics.

## AUTHOR CONTRIBUTIONS

**Seth Wenger:** Conceptualization (lead); data curation (equal); formal analysis (lead); methodology (equal); writing – original draft (lead); writing – review and editing (equal). **Ed Stowe:** Data curation (equal); formal analysis (equal); methodology (equal); writing – original draft (equal); writing – review and editing (equal). **Keith Gido:** Data curation (equal); methodology (equal); writing – review and editing (equal). **Mary Freeman:** Data curation (equal); methodology (equal); writing – review and editing (equal). **Yoichiro Kanno:** Methodology (equal); writing – review and editing (equal). **Nathan Franssen:** Methodology (equal); writing – review and editing (equal). **Julian Olden:** Methodology (equal); writing – review and editing (equal). **N. LeRoy Poff:** Methodology (equal); writing – review and editing (equal). **Annika Walters:** Methodology (equal); writing – review and editing (equal). **Phillip M. Bumpers:** Data curation (equal); methodology (equal); writing – review and editing (equal). **Meryl Mims:** Methodology (equal); writing – review and editing (equal). **Mevin Hooten:** Methodology (equal); writing – review and editing (equal). **Xinyi Lu:** Methodology (equal); writing – review and editing (equal).

## CONFLICT OF INTEREST

The authors declare no conflicts of interest.

## Supporting information


Table SI1
Table SI2

## Data Availability

Datasets and code for simulations and case studies are available on Zenodo: 10.5281/zenodo.5478098.
