# Supplementary material for: Simple statistical models can be sufficient for testing hypotheses with population time‐series data
Source: Ecol Evol. 2022 Sep 27;12(9):e9339. doi: 10.1002/ece3.9339 (PMC9514214; doi:10.1002/ece3.9339)
Supplement: Supplementary file 1 — Table SI1 Table SI2 [file ECE3-12-e9339-s001.docx]

**Supporting Information 1. Results of non-Bayesian models for case studies.**

Table SI1. Parameter estimates (means and standard errors) and performance scores for the non-Bayesian versions of the three model types for six fish species. “High flow” and “Low flow” are variables representing the number of high flow days and low flow days in the current year; “lag” indicates the same variable for the prior year. “Q” is the discharge on the day of sampling. Superscripts indicate support for hypotheses of the corresponding number (i.e., parameter estimates with the expected sign and 90% confidence intervals that do not overlap zero). R^2^ is the squared correlation between conditional model predictions and observations (a pseudo-R^2^).

| **Species** | **Model** | **High flow** | **High flow lag** | **Low flow** | **Low flow lag** | **Q** | **R^2^** |
| --- | --- | --- | --- | --- | --- | --- | --- |
| *Cyprinella callistia*  (Alabama shiner) | A | -0.61 (0.02)^1^ | -0.14 (0.01) ^2^ | -0.09 (0.01) | -0.03 (0.01) | -0.29 (0.01) | 0.37 |
|  | B | -0.31 (0.08) ^1^ | -0.06 (0.05) | 0.14 (0.06) ^3^ | -0.04 (0.06) | -0.34 (0.06) | 0.33 |
|  | C | -0.15 (0.11) | 0.35 (0.09) ^2^ | 0.29 (0.08) ^3^ | -0.33 (0.08) ^4^ | -0.28 (0.09) | 0.51 |
| *Macrhybopsis etnieri*  (Coosa chub) | A | -0.25 (0.02) ^1^ | 0.00 (0.01) | -0.09 (0.02) ^3^ | -0.06 (0.02) | 0.02 (0.02) | 0.04 |
|  | B | -0.14 (0.11) | -0.01 (0.07) | -0.05 (0.09) | -0.13 (0.08) | -0.05 (0.09) | 0.04 |
|  | C | -0.04 (0.15) | 0.06 (0.13) | 0.18 (0.12) | -0.14 (0.11) | 0.02 (0.13) | 0.05 |
| *Noturus leptacanthus*  (Speckled madtom) | A | -0.49 (0.09) ^1^ | -0.02 (0.03) | -0.09 (0.04) ^3^ | -0.04 (0.04) | -0.24 (0.05) | 0.11 |
|  | B | -0.69 (0.16) ^1^ | -0.08 (0.08) | -0.23 (0.10) ^3^ | 0.01 (0.09) | -0.24 (0.11) | 0.10 |
|  | C | -0.37 (0.15) ^1^ | 0.28 (0.13) ^2^ | -0.04 (0.12) | 0.04 (0.11) | -0.02 (0.12) | 0.14 |
| *Noturus* sp. cf. *munitis*  (Coosa madtom) | A | -0.32 (0.06) ^1^ | -0.17 (0.03) ^2^ | 0.04 (0.03) | -0.23 (0.03) ^4^ | 0.10 (0.04) | 0.06 |
|  | B | -0.23 (0.14) | -0.13 (0.10) | 0.14 (0.10) | -0.18 (0.09) ^4^ | 0.15 (0.10) | 0.05 |
|  | C | -0.06 (0.15) | -0.05 (0.13) | 0.38 (0.12) ^3^ | -0.33 (0.11) ^4^ | 0.36 (0.13) | 0.14 |
| *Percina nigrofasciata*  (Blackbanded darter) | A | -0.50 (0.03) ^1^ | -0.24 (0.02) ^2^ | -0.13 (0.02) ^3^ | -0.13 (0.02) ^4^ | -0.26 (0.02) | 0.32 |
|  | B | -0.55 (0.10) ^1^ | -0.24 (0.06) ^2^ | -0.17 (0.07) ^3^ | -0.15 (0.06) ^4^ | -0.44 (0.08) | 0.35 |
|  | C | -0.29 (0.14) ^1^ | 0.36 (0.13) ^2^ | -0.14 (0.11) | -0.12 (0.11) | -0.42 (0.12) | 0.30 |
| *Percina palmaris*  (Bronze darter) | A | -0.48 (0.04) ^1^ | 0.00 (0.01) | -0.08 (0.02) ^3^ | -0.14 (0.02) ^4^ | -0.41 (0.02) | 0.23 |
|  | B | -0.41 (0.11) ^1^ | 0.05 (0.06) | 0.03 (0.08) | -0.15 (0.07) ^4^ | -0.36 (0.08) | 0.21 |
|  | C | -0.20 (0.12) | 0.59 (0.11) ^2^ | 0.21 (0.10) ^3^ | -0.34 (0.09) ^4^ | -0.27 (0.10) | 0.51 |

Table SI2. Parameter estimates (means and standard errors) and performance scores for the non-Bayesian versions of the three model types for six mammal species. “Precipitation” and “Time-since-burning” are variables representing the amount of precipitation in the preceding year and the number of years since prescribed burns occurred at the site. Superscripts indicate support for hypotheses of the corresponding number (i.e., parameter estimates with the expected sign and 90% confidence intervals that do not overlap zero). R^2^ is the squared correlation between conditional model predictions and observations (a pseudo-R^2^).

| **Species** | **Model** | **Precipitation** | **Time-since-burning** | **R^2^** |
| --- | --- | --- | --- | --- |
| *Microtus ochrogaster* Prairie vole (herbivore) | A | 0.77 (0.05) ^1^ | 0.39 (0.08) | 0.33 |
|  | B | 0.90 (0.19) ^1^ | 0.43 (0.20) | 0.34 |
|  | C | 0.37 (0.10) ^1^ | 0.04 (0.10) | 0.10 |
| *Sigmodon hispidus*  Hispid cotton rat (herbivore) | A | 0.04 (0.06) | 0.33 (0.09) | 0.08 |
|  | B | -0.10 (0.22) | 0.56 (0.25) | 0.07 |
|  | C | -0.10 (0.09) | 0.01 (0.10) | 0.01 |
| *Blarina hylophaga*  Elliot’s short-tailed shrew (insectivore) | A | 0.64 (0.05)^2^ | -0.05 (0.08) | 0.27 |
|  | B | 0.71 (0.13) ^2^ | 0.16 (0.15) | 0.25 |
|  | C | 0.50 (0.10) ^2^ | 0.01 (0.10) | 0.19 |
| *Peromyscus leucopus* White-footed mouse (omnivore) | A | 0.00 (0.03) | 0.30 (0.03) | 0.53 |
|  | B | 0.05 (0.08) | 0.37 (0.16) | 0.53 |
|  | C | 0.06 (0.08) | 0.01 (0.08) | 0.01 |
| *Peromyscus maniculatus* Deer mouse (omnivore) | A | 0.05 (0.03) | -0.40 (0.09)^4^ | 0.27 |
|  | B | 0.08 (0.06) | -0.43 (0.13) ^4^ | 0.26 |
|  | C | 0.02 (0.07) | -0.04 (0.08) | 0.00 |
| *Reithrodontomys megalotis* Western harvest mouse (granivore) | A | 0.01 (0.05) | -0.20 (0.07) ^4^ | 0.03 |
|  | B | 0.01 (0.13) | -0.01 (0.19) | 0.01 |
|  | C | 0.03 (0.09) | -0.01 (0.09) | 0.00 |
